# Reasons for Declining to Enroll in a Phase I and II HIV Vaccine Trial after Randomization among Eligible Volunteers in Dar es Salaam, Tanzania

**DOI:** 10.1371/journal.pone.0014619

**Published:** 2011-02-16

**Authors:** Edith A. M. Tarimo, Anna Thorson, Thecla W. Kohi, Muhammad Bakari, Fred Mhalu, Asli Kulane

**Affiliations:** 1 Division of Global Health, Department of Public Health Sciences, Karolinska Institutet, Stockholm, Sweden; 2 Department of Nursing Management, Muhimbili University of Health and Allied Sciences, Dar es Salaam, Tanzania; 3 Department of Internal Medicine, Muhimbili University of Health and Allied Sciences, Dar es Salaam, Tanzania; 4 Department of Microbiology and Immunology, Muhimbili University of Health and Allied Sciences, Dar es Salaam, Tanzania; The George Washington University Medical Center, United States of America

## Abstract

**Background:**

Recruitment, enrollment and retention of volunteers in an HIV vaccine trial is important in the efforts to ultimately develop a vaccine that can prevent new HIV infections. Following recruitment, some randomized individuals decline to be enrolled in an HIV vaccine trial. The reasons for such a decision are not well known. This article describes why individuals who were randomized in a phase I and II HIV vaccine trial in Dar es Salaam, Tanzania declined to be enrolled.

**Methods:**

Face-to-face interviews were conducted with 14 individuals (7 men and 7 women). Repeated readings of the 14 interview transcripts to look for reasons for declining to enroll in the trial were performed. Data was analyzed using the content analysis approach.

**Results:**

Informants expressed fear of the outcome of an experimental HIV vaccine in their lives. Unlike women, some men were concerned over the effect of the vaccine on their reproduction intentions. Women were concerned about the unknown effects of the vaccine in their bodies. Also, to a large extent, informants faced resistance from significant others such as fiancées, parents, relatives, and friends. Women were influenced by their potential intimate sexual partners; men were forbidden by their parents, and mothers had the most influential opinion.

**Conclusions:**

Fear of the negative outcome of an experimental vaccine and resistance from significant others are the main reasons for declining to enroll in the HIV vaccine trial among eligible volunteers after randomization. The resistance from the significant others provides valuable guidance for designing future trials in Tanzania; for example, expanding the HIV vaccine trial education to the general population from the onset of the trial design.

## Introduction

Throughout the world, only one HIV vaccine candidate has shown a modest efficacy in a phase III trial [1]. Multiple trials are needed to develop an eventual effective HIV vaccine. However, conducting trials is difficult for several reasons including challenges experienced during recruitment, enrollment, and retention of study participants [2], [3], [4], [5], [6], [7], [8], [9], [10]. In previous studies, participants were not ready to take part in an experimental HIV vaccine because of fear of becoming infected with the HIV virus and mistrust of governments conducting the trials [4], [7], [8], [10], [11]. Women were concerned about the potential effects of HIV vaccine trial on their reproductive health [10], [12], [13]. Also they sensed that taking part in the trial would bring conflicts in their parental roles, negotiating safe sex with male partners, worries about being stigmatized, and being discriminated against [10].

In order to increase the retention of future volunteers, it is important to understand reasons that influence eligible individuals not to enroll in HIV vaccine trials. Globally, few studies have focused on why people decline to enroll in HIV vaccine trials [6], [14]. In these studies, trial duration, concerns about false-positive HIV test results, side effects and negative reactions from partners were commonly cited as reasons for declining to enroll in the HIV vaccine trials. In one study, trial duration was a factor that influenced individuals not to complete follow-up visits during the trial [5]. These studies were conducted in the high income countries. The sub-Saharan African countries have the highest HIV infection rates and disease burden, but fewer HIV vaccine trials have been conducted than in the United States and Europe [15], [16]. Conducting trials in low income countries is equally important given the burden of HIV infection rates [17], and retention of those who volunteer for the trials is therefore crucial to maximize use of resources [18]. Little is known from Africa about why individuals enroll in HIV vaccine trials and subsequently withdraw.

Tanzania is among the low income countries conducting Phase I and Phase II HIV vaccine trials [16]. During the recruitment process, some of the randomized eligible volunteers declined just before the first vaccination [actual enrollment]. The term ‘decline’ in this study is defined as an act of a screened, eligible and randomized volunteer not showing up to receive the first vaccine [DNA or placebo] dose within 30 days after randomization. According to the trial plan, the first vaccination was scheduled 14 days after randomization. Therefore, the purpose of this study was to understand why some individuals who were randomized in a Phase I and II HIV vaccine trial (HIVIS03) in Dar es Salaam, Tanzania, subsequently declined. This study produces knowledge of reasons for declining to enroll in the HIV vaccine trial by using the content analysis approach [19].

## Materials and Methods

### Ethics statement

This study draws material from a randomized double-blind phase I and II HIV vaccine trial research project conducted among healthy volunteers in Dar es Salaam, Tanzania [16]. The project was approved by the National Institute for Medical Research (NIMR) Ethics Committee which offered a letter(s) with reference number NIMR/HQ/R.8a/Vol.IX/410. This approval was for the whole HIV Vaccine Trial project protocol that included follow up of the study participants and documentation of the reasons for withdrawal from the study. In the present study, after describing the purpose of the study, the first author reminded the potential informants of their signed consent before they declined that included agreement to be followed up. All potential informants consented for tape-recording of the interviews, although one interview was not recorded because the environment was too noisy. Informants were not paid for their participation.

Note: In detail, potential volunteers signed informed consent, part 1 before they were screened for enrollment in the trial. The screening involved: clinical history and examination; HIV counselling and testing; laboratory tests that included blood tests to screen for syphilis and hepatitis B infections, haematology and clinical chemistry as well as urine collection for pregnancy test among females. Two weeks later, they signed informed consent, part 2 to confirm their enrollment in the HIV vaccine trial and follow up if they skipped the planned schedules. This procedure of signing two parts of the informed consent was stated in the original project protocol. Also, during this second visit all laboratory results in line with fulfilling the inclusion criteria were reviewed and the volunteer was assessed to make sure that he or she understands the objectives of the study.

### Informants

All informants participated in a series of educational sessions on HIV, AIDS and HIV vaccine trial concepts and procedures that were conducted from 2006 to 2007 among potential volunteers for the HIV vaccine trial [13], [20].

**Figure 1:**
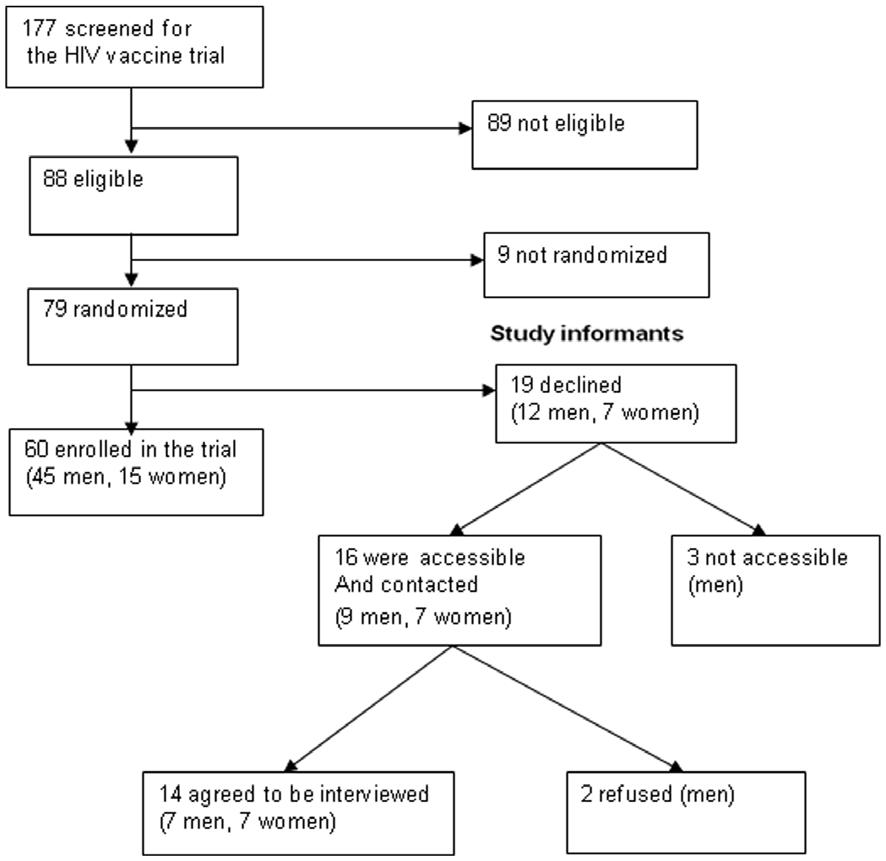
Recruitment of the study informants.

A total of 177 individuals were screened for the trial between February 2007 and February 2008. Of these, 89 (50.3%) were ineligible on a medical basis after thorough medical and laboratory screenings were done. Nine (5.1%) were eligible but were not randomized for the study because the enrollment had been closed; 79/177 = 46.6% were eligible and were randomized to enter the trial. However, 19/79 = 24.1%, 12 men and seven women, declined to enroll after randomization. Thus, 12/57 = 21% men and 7/22  = 32% women declined enrollment after randomization. Sixteen out of 19 (9 men and 7 women) were accessible and were contacted 1–23 months after declining for this follow up study. The first author contacted the individuals through their mobile phones, briefed them about the aim of the present study, and asked for their willingness to discuss with the researcher (first author). Of the 16 individuals, 2 men refused to take part in this study without explanations. The rest, 14/19 = 74% agreed, were accessible, chose to meet the researcher at their workplace and participated in this study fully (See figure 1).

### Study design

While informants were purposively selected because they are ‘information rich’ for the phenomenon of interest [21], the sample was convenient in the sense that it was all inclusive of those who declined to enroll in the trial and accessible to take part in this study.

### Data collection

#### Face-to-face interview

Fourteen informants were interviewed about the reasons for declining to enroll in an HIV vaccine trial after randomization. The first author who was also part of the recruitment team conducted all interviews in privacy within the individuals' workplace, using an interview guide. The guide consisted of the following statements: ‘I understand you are among the volunteers who were randomized in an HIV vaccine trial; however, later you decided not to continue with the planned visits for the vaccinations. Can you explain to me the reasons for not continuing with the scheduled vaccinations?’ This was followed by probing set of questions according to the responses. After the first 8 interviews, the guide was expanded to include their suggestions to improve future recruitments for HIV vaccine trials. Thirteen interviews were audio-taped, lasted for 10–30 minutes each, and were conducted between April 2007 and November, 2009. One interview was not tape-recorded because the environment was noisy.

### Analysis

A research assistant transcribed the audio-tapes word for word. Another research assistant translated all transcripts from Kiswahili, Tanzania's national language to English. The translations were checked by the first author (EAMT) who speaks both languages. EAMT listened to the audiotapes and at the same time read all transcripts to ensure that there were no parts of the discussions lost during transcription. She repeatedly read all interview transcripts to understand what each participant communicated about the topic. The data was analyzed using a content analysis approach as suggested by Graneheim [19], and the results were mainly manifest in content. At the beginning, the meaning of each participant's response about declining was coded and written on the margin of the transcript. The text was divided into sentences and paragraphs (meaning units) that were condensed, abstracted and labelled with codes (see example in Table 1). The codes were sorted manually into subcategories, categories, and one theme emerged. The categories and subcategories were discussed, negotiated and revised by the first and the last author [AK]. We used quotes to ensure that informants' views are reflected in the paper, and for the purpose of this interview, informants' sex and serial numbers are assigned in the text to protect their identity.

**Table 1:** Example of a meaning unit, a condensed meaning unit and codes from content analysis of reasons for not enrolling in an HIV vaccine trial.

Meaning unit	Condensed meaning unit Description close to the text	Codes
I didn't understand, we [researchers and I] don't even know for how long that vaccine will stay in the body! You set insurance of two years, but I may get any problem after two years and think that, it is because of that vaccine. I will go to hospital and given panadol and asprin and will not get proper treatment!	Researchers and I don't understand how long the vaccine will stay in the body; I may get problems when insurance is over and will be not get proper treatment	Fear of side effectsUnsure of health services after the trialPrerequisite for a prolonged health insurance

## Results

### Description of the informants

Seven men and seven women were interviewed. Their ages ranged between 20–38 years, with an overall mean age of 28 years. Five men identified themselves as single and without children. Two men were married, and two had children during data collection. Three of the women were married with one to two children each, and one was a single parent. Thirteen of the informants had attended four years of secondary education and one had completed seven years of primary education.

### Theme and categories

The reasons for declining to continue participation in the HIV vaccine trial as described by informants in this study are under one theme: ‘Perceived fear towards enrolling in an HIV vaccine trial’. This theme included two categories. The first category, ‘Personal fear of the HIV vaccine’, brings attention to the possible side effects that would harm the informants in different ways and at different periods. The second category, ‘Resistance from the significant others’, highlights discouragements from other people who pointed to the belief that the vaccine is harmful and would interfere with social bonds.

### Personal fear of the HIV vaccine

#### Potential side effects of vaccine in the reproduction continuity

Even though all participants expressed enthusiasm and free will to enroll in the HIV vaccine trial at the time of consent, most of them declined later because of fear. Some men stated that they intended to continue with the trial, but they had to stop before receiving the vaccine because of fear of the effect that the vaccine could have on their reproduction intentions. They were worried about a series of rules on ‘dos and don'ts’ that were issued by the researchers. They believed the vaccine might be harmful in their reproduction continuity, and the trial would mess up their marriage relationship. One participant expressed the fears as follows:

…they [researchers] presented it in this way: ’you are not supposed to do sexual intercourse without a condom for a certain period. Then a woman should not conceive or get pregnant. I mean you are not supposed to give birth in that period. So, we perceived that when they are giving this to us and stay in our body for a long time it will cause harm… That is the main reason for us to discontinue, and saying that ‘even if you will be given that vaccine will be there a feeling in your marriage relationship? It will only bring disturbance!’ (Informant 3, man)

A newly married man was mostly concerned with the trial rules that seemed to interfere with his marriage intentions. He was worried about the rules, given that he just got married, not having a child as yet, and the vaccine was on an experimental basis. He decided to postpone enrolling and gave priority to having a child. He narrated:

First, it was the vaccine on trial, and we were told that if we accept to participate in that program we are not supposed to engage in penetrative sexual intercourse with any woman for a year to avoid its effects in pregnancy. At that time, I was doing another attempt in order to get a child! (Informant 12, man)

On the contrary, women stated that pregnancy restriction was not a problem, but they declined because of other types of fears. One woman confidently understood that enrollment in the trial cannot interfere with her reproduction capacity because she trusted the information given about the trial safety on reproduction. She believed she could get a child after the trial. She reflected:

We asked in one of the seminar that if a woman participates in the vaccine what happens … They [researchers] said there will be no problem… but in this period she is not supposed to conceive. In that case, I have seen that I will get a child afterwards [after completing the vaccinations in the trial] (Informant 11, woman)

Another woman with two children added that, her reason for declining was not fear of vaccine's effect on the reproduction, but other possible side effects:

I didn't have that worry of being infertile because of the vaccine … I may get another child if I wish … but my worry is that I don't know what will be the side effects of that vaccine in the body! (Informant 14, woman)

In addition, the perceived negative effects of the HIV vaccine trial were multiple as described in the following section.

#### Possibilities of unknown negative effects of the vaccine in the body

Some women said that they became nervous about the vaccine's side effects in different ways. One woman declared that she was in the forefront in the HIV vaccine trial study, motivating colleagues to take part, but she stepped down after the death of her sister [the sister was not in the trial]. She stated that, the knowledge she gained about the trial could not overcome her fear after the funeral of her late sister. Nevertheless, she felt impassive in her fear:

I did all the screening procedures. Everything was good and I understood everything … my sister passed away while I was getting prepared to get the first vaccine …. Surprisingly after the funeral, I was tense and deeply felt that I should not continue to enroll in the trial … (Informant 8, woman)

When probed to explain more, she insisted that she couldn't understand what was happening in her body [mind]. She elaborated:

Do you know something that makes you cautious not to do something for no obvious reason? That was what happened to me… my fear is inside my heart. I just feel I cannot continue with the trial! (Informant 8, woman)

Another woman who was also preparing to get the first vaccination described her fear after seeing a colleague (man) excessively vomiting a day after the vaccination. She heard rumours all over the workplace: ‘Those are the vaccine's side effects! [Some colleagues insisted]’. Although, she recalled that after medical consultation (not at the trial clinic), it was clear the cause of the vomiting was excessive alcohol taking, she did not believe in that explanation. She was worried, but she felt responsible and need to be nice to the trial team:

… a nurse from Muhimbili [study nurse at the trial clinic] called me, and I had nothing to say except promising that I would go to the clinic. I don't know if it would be appropriate to tell that I would not go! (Informant 7, woman)

Even though she had already made up her mind not to continue with the trial, she experienced guilt. While recalling the politeness of the trial team, she said:

You know what I am ashamed of is to meet such incredible people at the clinic; stating that I don't want to proceed with the trial! I remember doctors, nurses, and counsellors; the way they handled me so friendly with a cup of tea with milk ….” No, but my heart doesn't encourage me to continue [proceed with the trial] … (Informant 7, woman)

On the vaccination day, another woman reversed her decision of receiving the vaccine because of personal fear of not involving her mother. Thus, she wanted to postpone the vaccination in order to consult her mother first. However, she felt that the service providers at the trial clinic did not support her concern accordingly. So she quit and explained that her mother was supposed to know before she received the vaccination. She insisted that her mother was a very important person in her decision. She explained:

When I told them [service providers at the trial clinic] that I wanted to talk to my mother first before I get the vaccine, they became impolite …I mean they failed to convince me at the last point … I told them that I'm going to talk to my mother because she is second after God. So, if she accepted, I would accept too (Informant 9, woman)

Each of these women expressed fear in different ways, but importantly, the fears reversed their decisions to enroll in the HIV vaccine trial.

#### Uncertainty about the insurance during and after the trial

Informants continued to fear of vaccine outcome in both short and long terms and in case the trial fails and causes harm. They were not satisfied with the information given about the insurance, and particularly, the length of health insurance that was planned to end after the trial. They doubted about their future in case of negative outcomes of the trial. One informant highlighted the uncertainty about the insurance:

They [researchers] are saying this program will end next year… if it [vaccine] brings side effects and the health insurance has expired, perhaps I will be a victim. Now, I don't know which side will I be, which group will I belong to? You see, things like these can make a person doubt about it (Informant 3, man)

Another informant believed the information about the trial safety and insurance was not convincing at all. She feared that the vaccine could have effects that were not known to anybody, even to the researchers themselves. She imagined that the trial could possibly involve health related risks and felt the need for an extension of the planned health insurance:

I didn't understand …., we [researchers and I] don't even know for how long that vaccine will stay in the body! You set insurance of two years, but I may get any problem after two years and think it is because of that vaccine …insurance [continuation of health insurance] is very important (Informant 14, woman)

In addition, this informant expressed fear of joining in the trial; the trial that did not even guarantee life insurance. Her worry was exacerbated by the possibility of death because of vaccine side effects and leaving the family unsupported. She felt that life insurance was among the prerequisites for her to enroll in the trial. She stated:

Even if I get affected to death, then I leave the family. My family should benefit to some extent such as children getting school fees, but they [trial team] said there is no such kind of insurance; this is just the issue of voluntarism. That thing really created more doubts (Informant 14, woman)

Informants insisted on the need for adequate health and life insurance for the volunteers in the trial. Also, some claimed that the paying of 20USD to cover travel and time cost which was allocated for the enrolled volunteers was not enough. One informant suggested for an increase to cover special meals in case a volunteer gets sick during the trial.

#### Mistrust of the researchers' intentions

Somehow, informants were suspicious about the researchers' intentions given that they were informed that the vaccine might have unknown side effects. However, they did not believe the safety part of the vaccine although it was explained by the researchers during the seminars. One informant believed that the researchers were afraid of taking part in the trial because of uncertainty about the trial:

It may have negative effects in the future. You [researchers] insisted that the vaccine has no side effects [serious side effects] but it is not true. One day I asked one of you who facilitated the seminar that ‘who gets an HIV vaccine among you?’ They said ‘we are not allowed to get that vaccine because we are service providers.’ Don't you see that you are avoiding something? (Informant 14, woman)

Overall, these personal fears were diverse, and each informant had a way of expressing his or her concern(s) which were either single or multiple according to individuals' descriptions.

### Resistance from significant others (fiancée, parents, relatives and colleagues)

#### Uncertainty about the close relationships

Although most informants expressed enthusiasm to enroll in the trial; they realized that a final decision had to receive the approval of significant others. They voiced that their significant others did not trust the content of the HIV vaccine. Therefore, they were discouraged from continuing with the trial. Under such circumstances, informants were forced to weigh whether to enroll in the trial against the opinions of the significant others. Consequently, both men and women feared breaking the existing social bonds because of enrolling in the trial. Women were mostly influenced by the intimate sexual partners. One woman was indirectly warned by her fiancée and she couldn't force him. She narrated:

It doesn't mean that I don't want to proceed with vaccination process. My fiancée is the cause because when I was about to get the first vaccination, I described to him… he kept on saying: ‘wait a little bit, I have to think critically and then I will let you know’. And this has been a long time since he commented… That is the main obstacle (Informant 2, woman)

Another informant explained that she declined because her fiancée forbade her to enroll straight away. Although she felt bad about it she could not force him in order to protect their marriage intentions. She said:

…my fiancée did not accept it completely! And he warned if I enroll in the trial our relationship would end; even though he had already paid a dowry, he would cancel our marriage plans… I felt bad because I had already committed myself with that relationship and I saw there is no need to force him (Informant 11, woman)

Another woman (informant 5), who had a six-year-old child stated she was forbidden by her husband to enroll when she was just about to receive the first vaccination. She recalled that her husband supported her in the beginning but not in the end. She suspected the husband had desired to have a second child.

#### Responsibility to care for parents

Unlike women; men expressed the need for consensus from their parents to continue with the enrollment in the trial. However, they stated that they were cautioned and reminded about their responsibility to the aging parents. They received series of warnings from their parents including mistrust of the imported vaccine. Under such opinions and warnings, young men hesitated to argue with their parents. Finally, they realised that enrolling in the vaccine trial was not the best. The following quote reflects the dialogue:

I told my parents but they told me that: ‘Do you know we are getting old now? … … wazungu [white skinned people] brought these things [vaccines] to you, and you are searching for death …You have to tell me the day when you are going to get that vaccine, I mean you have to tell me! Don't go there before telling me’ [the mother warned]. She insisted that I should not go without telling her. I didn't know her intention of doing that (Informant 3, man).

Also, other men realised that their mothers were suspicious about possible negative outcome of the trial; the outcome that could distort the expected responsibilities from the sons. They felt responsible and the need of ‘being the insurance for their parents’ despite the fact that they were interested to enroll in the trial. The following discourse explains:

When I told my mother about that [vaccine trial], in brief she was shocked! … On top of that I'm the only son remain in our family; the rest passed away… She insisted I should stop where I reached. That means I should stop. I asked her why? She said: ‘that is what I am saying, if you are going against, it will be your decision and what I have told you, that is it’. So, I thought of that… That was the end of the exercise, but I was not happy to stop there (Informant 13, man)

They added that parents were against their intention to enroll in the trial, and were obliged to follow their opinions:

… They [parents] told me that: ‘As your parents, we don’t agree with your idea…; if you don’t listen to us; fine’… They cautioned me not to continue with my mission and insisted: ‘we ask you to discontinue with those things, and if you object, just go and whatever happens will be up to you’. Therefore, I had to listen to my parents (Informant 6, man).

In addition they faced resistance from their fathers. A married man with two children described how he was convinced by his father. Following his father's advice, he decided to drop his intention to enroll in the trial at the last minute. He said:

What hindered me is him [father] … he listened to it and then said ‘it is good but most of the time that kind of exercise [trial] brings effects to people in future… So long my father told me about that I saw that there is no reason to participate on that vaccine. That is why I decided to quit … (Informant 10, man)

Other men described how they struggled to sell the idea of the trial to their families, but all efforts ended in vain. One informant recognized the importance of his parents as the main advisors, and so had to obey their opinions despite his enthusiasm to enroll in the trial:

It is parents who advised me. I was ready to get that vaccine but my family queried: ‘Can't this vaccine cause HIV infection in your body?’ I told them, it will not, but my mother warned me: ‘You should stop, stop continuing with that’… As you know, they [parents] are core advisors especially to me at this time (Informant 1, man).

Another man expressed that the family, particularly his brother did not support his decision to enroll in the trial at all. He suspected that the brother feared that he would deliberately die because of introducing a virus through the vaccination:

… I explained it to my relatives but they didn't believe it! … They started telling me that ‘you protect yourself for a long time and then you go to be infected with HIV!’ … My brother opposed and said: ‘you are still young; you are going to lose your life for such things!’ I explained but he did not understand (Informant 4, man)

Generally, the collective support sought from the close members of the family reversed the informants' mind-set. At moments of increased different opinions, informants simply lost control over their enthusiasm and decision to enroll in the trial.

#### Discouragement from colleagues and friends

In addition, informants reported discouragement from outside the family; especially, from the colleagues and friends who posed queries about the safety of the HIV vaccine trial. Such discouragements reversed informants' decisions to continue with the trial, and given that they had no evidence of seeing a volunteer who had received a similar kind of vaccine. They reflected on the warnings from colleagues who warned them right after the HIV vaccine seminars that enrolling in a vaccine trial will be dangerous in the future. This opinion forced some informants to decline.

Informants recognised that most of the colleagues who were discouraging them were not exposed to the HIV vaccine trial education sessions. Nevertheless, they were forced to weigh their decision against opinions of others because they relied on them for socialisation. Thus, they illustrated moments of uncertainty whether to join in, but the influence of friends overpowered their decision.

## Discussion

This study illustrates that informants declined to enroll in the HIV vaccine trial because of single or multiple reasons. They feared the potential side effects of an experimental vaccine in their lives, such as interference with reproduction intentions; possibilities of harmful effects, to the extreme death in absence of life insurance. This is especially so in the absence of a life-time health insurance guarantee. They are doubtful about the researchers' trustworthiness in the vaccine trial. Also they declined largely because of resistance from the significant others. Therefore, the reasons for declining to enroll in an HIV vaccine trial are seen as a combination of fears.

The fear of an experimental HIV vaccine on reproductive intentions could be due to the fact that these informants were young and had already planned to start families with children before the trial came in. Despite the information given about the trial safety, they dared not to postpone having children because of fear of possible irreversible side effect of the vaccine in reproduction. However, this fear was raised by more men than women, implying that women understood that the trial was safe in their reproductive capacity. On the contrary, previous studies show that women are concerned about the effect of HIV vaccine trial on their reproduction [10], [12]; and the need to delay pregnancy during trial had a larger effect on willingness to take part in the HIV vaccine trials [22].

In this study, the fear of harmful effects from the trial could be due to concurrent incidents (a colleague vomiting after the trial, and an experience of losing a sibling, not connected to trial though) which posed threats to the informants just before they got the first vaccination. In the first incident, these co-workers [informant and the volunteer] must have discussed their participation in the HIV vaccine trial voluntarily. In a previous study, potential volunteers reported to seek opinions about their decision to volunteer in the HIV vaccine trial from the significant others such as co-workers [13]. Thus, the two workers reported here must have known each other through such interactions and participation in the recruitment seminars. The informant in the second incident was seriously worried by death of the sibling. Mentally, these informants might have been in doubt even before the incidents, and that the incidents just aggravated their decisions of not to enroll in the trial. Fear of unknown as portrayed by one of the informants can be interpreted as fear of death; death that is viewed as likely to visit the same family because of uncertainty about vaccine safety. The novelty of the experimental vaccine, as noted elsewhere [14], can aggravate the fear.

The resistance from the significant others could be associated with lack of awareness about HIV vaccine trials in the general population. The significant others believed that, HIV vaccine trials could affect the informants in different ways. The most important concern was the possibility that the candidate vaccine could cause death; death that could break the established social support networks. This concern may be due in part to the fixed social support networks in African families [23]. In a previous study among police officers, respondents significantly stated that they would share their intent to volunteer in an HIV vaccine trial with significant others so that they may take care if they suffer from side effects [20]. Conversely, in the present study, the discouragement from the parents was the possibility that their sons could die because of enrolling in the trial and end the dependence chain. This fear signifies the importance of young men for the aging parents in a Tanzanian context as stated earlier by potential volunteers for a phase I and II HIV vaccine trial [13]. Partly, the fear could also be fuelled by experiences of death of young adults due to AIDS in sub-Saharan African countries [24]. Thus, the concerns pointed out by the parents indicate worries of deliberately losing young adults who would otherwise be guarantees of support in their old age. Interestingly, more men consulted parents, and more women consulted sexual partners for approval to enroll in the trial. This discourse provides a clue on influences of specific types of significant others on the potential volunteers in future trials. However, such influence confronts women in their struggle for own decision to participate in the HIV prevention strategies.

### Limitations

The results of this study cannot be generalised beyond the context we studied, but can be transferred to similar contexts. Also, given that almost all those who declined to enroll in the HIV vaccine trial were interviewed implies that, there was no room for saturation [25] as would have been the case for a traditional qualitative sample. The interview sessions reflect the number and details of the reasons for declining to enroll in the trial provided by the informants, and most of the informants were interviewed 1–3 months after randomization and withdrawal from the study. Although, four informants were contacted and interviewed 20–23 months after randomisation and withdrawal from the study, they used almost the same amount of time during the interview. On the one hand this delayed contact could have influenced the recalling of reasons for declining. However, the probing nature of qualitative interviews enabled the researcher to gain in-depth description of the stated reasons. Furthermore, to some, the long period between randomisation and this follow up interview might have enabled the informants to reflect and recall the key reason(s) for declining. Nevertheless, we can not tell the impact of this delayed contact on reasons given from the individual interviews because some of the reasons given by the four informants were similar to what earlier informants had shared.

Although it could be difficult to access most people who decided not to participate in the HIV vaccine trial for such interviews, we managed to access our study informants through a well established cohort in the police force. In this cohort, a group of individuals voluntarily formed a core group to educate others about HIV, AIDS and other health related issues which were supported by experts from the trial team. All informants in this study happened to be part of the core group, and they remained active in the core group despite their decision to decline from the trial. Thus, access was not a problem for those who were still working in Dar es Salaam region.

We could not reach three men who declined because they were relocated in other work places, outside Dar es Salaam. In addition, two refused to participate in the study without providing explanations. As researchers, we were curious to know what their reasons were, but ethically it was not acceptable to enquire about their reasons.

The interval between randomization and vaccination might have facilitated the potential volunteers to enquire further advice from the significant others that lead to changing their decision to enroll in the trial. Although potential volunteers were informed from the start to share their decision with the significant others if they wished to, they abruptly decided to consult them after randomization. On one hand, it was their ethical right to do so at any time. However, it would have been wise to perform this consultation from the beginning to maximize use of the resources.

Also the procedure of giving vaccination 14 days after randomization that was employed at this site might have facilitated the informants to change their decision to enroll in the trial at a later stage after recruitment. However, this could also have led to self-exclusion of volunteers who were not yet firmly decided on enrollment despite being recruited by study staff. The trial team comprised of trained health care providers (Doctors, Nurses, Nurse Counsellors, Laboratory technologists and Support staff) with training and Certification in Good Clinical Practice (GCP) or Good Clinical Laboratory Practice (GCLP). Additionally, Standard Operating Procedures specific for conducting the HIV vaccine trial were adhered to. Despite the shortfalls, the knowledge gained and being shared appears important for the design of future HIV vaccine trials in similar contexts.

### Implications

In this study, informants suggested that HIV vaccine trial education should target the general population rather than limiting education to a specific group of individuals. They emphasized that, the best settings for HIV related education could be the common meeting places such as recreation centres. They also highlighted the need to involve significant others, example relatives of those who enroll in the trial from the onset as stated by one of the informant:

“In the meetings, if I could come with my close relative of whom I believe if he or she gets such education and accept, both we would agree” (Informant 13, man).

Retention of randomised volunteers in HIV vaccine trials is important to maximise use of resources in conducting trials. These findings call for HIV vaccine trial implementers to consider the influences of significant others when recruiting volunteers in HIV vaccine trials and clear the doubts. Also, trial implementers should encourage extensive discussion about social issues with the potential volunteers to enhance disclosure of potential barriers from the surrounding communities. The insurance facts and personal fears need to be clarified through extensive counselling. For example, the duration of the vaccination contents in the body and safety in reproduction continuity should be transparent to clear the doubts. This study contributes important knowledge for future recruitment of volunteers for HIV vaccine trials in Tanzania and in other similar contexts.

### Conclusions

This qualitative study illustrates that the main reasons for declining to enroll in the HIV vaccine trial after randomisation are fear of harmful effect of the vaccine in the trial and resistance from uninformed or poorly informed significant others. We are witnessing the complexity of decision making within the social networks in times of doubt. Within these social networks, informants are bound to share decisions with ones they depend on for social support. Thus, it is what the informants had doubts on that impacted on their final decision. The faced resistance from significant others suggests the need to involve them during the recruitment of volunteers in the future as suggested by the informants themselves. In the light of these findings, trial implementers can oversee possible factors that can influence retention of those who will volunteer in the future trials.

## References

[1] Rerks-Ngarm S, Pitisuttithum P, Nitayaphan S, Kaewkungwal J, Chiu J (2009). Vaccination with ALVAC and AIDSVAX to prevent HIV-1 infection in Thailand.. N Engl J Med.

[2] Brown-Peterside P, Chiasson MA, Ren L, Koblin BA (2000). Involving women in HIV vaccine efficacy trials: lessons learned from a vaccine preparedness study in New York City.. J Urban Health.

[3] Brown-Peterside P, Rivera E, Lucy D, Slaughter I, Ren L (2001). Retaining hard-to-reach women in HIV prevention and vaccine trials: Project ACHIEVE.. Am J Public Health.

[4] Buchbinder SP, Metch B, Holte SE, Scheer S, Coletti A (2004). Determinants of enrollment in a preventive HIV vaccine trial: hypothetical versus actual willingness and barriers to participation.. J Acquir Immune Defic Syndr.

[5] de Bruyn G, Hudgens MG, Sullivan PS, Duerr AC (2005). Participant retention in clinical trials of candidate HIV vaccines.. J Acquir Immune Defic Syndr.

[6] Harro CD, Judson FN, Gorse GJ, Mayer KH, Kostman JR (2004). Recruitment and baseline epidemiologic profile of participants in the first phase 3 HIV vaccine efficacy trial.. J Acquir Immune Defic Syndr.

[7] Mills E, Cooper C, Guyatt G, Gilchrist A, Rachlis B (2004). Barriers to participating in an HIV vaccine trial: a systematic review.. Aids.

[8] Newman PA, Duan N, Roberts KJ, Seiden D, Rudy ET (2006). HIV vaccine trial participation among ethnic minority communities: barriers, motivators, and implications for recruitment.. J Acquir Immune Defic Syndr.

[9] Newman PA, Duan N, Rudy ET, Anton PA (2004). Challenges for HIV vaccine dissemination and clinical trial recruitment: if we build it, will they come?. AIDS Patient Care STDS.

[10] Rudy ET, Newman PA, Duan N, Kelly EM, Roberts KJ (2005). HIV vaccine acceptability among women at risk: perceived barriers and facilitators to future HIV vaccine uptake.. AIDS Educ Prev.

[11] McGrath JW, George K, Svilar G, Ihler E, Mafigiri D (2001). Knowledge about vaccine trials and willingness to participate in an HIV/AIDS vaccine study in the Ugandan military.. J Acquir Immune Defic Syndr.

[12] Mills E, Nixon S, Singh S, Dolma S, Nayyar A (2006). Enrolling women into HIV preventive vaccine trials: an ethical imperative but a logistical challenge.. PLoS Med.

[13] Tarimo EAM, Thorson A, Kohi TW, Mwami J, Bakari M (2010). Balancing Collective Responsibility, Individual Opportunities and Risks: A Qualitative Study on how Police Officers reason around volunteering in an HIV vaccine trial in Dar es Salaam, Tanzania.. BMC Public Health.

[14] Newman PA, Daley A, Halpenny R, Loutfy M (2008). Community heroes or “high-risk” pariahs? Reasons for declining to enroll in an HIV vaccine trial.. Vaccine.

[15] Esparza J, Osmanov S, Pattou-Markovic C, Toure C, Chang ML (2002). Past, present and future of HIV vaccine trials in developing countries.. Vaccine.

[16] International AIDS Vaccine Initiative (2009). http://www.iavireport.org/trials-db/Pages/default.aspx.

[17] UNAIDS (2009). AIDS epidemic update..

[18] SAAVI (2009). HIV Vaccine Info-line 080 VACCINE..

[19] Graneheim UH, Lundman B (2004). Qualitative content analysis in nursing research: concepts, procedures and measures to achieve trustworthiness.. Nurse Educ Today.

[20] Tarimo EA, Thorson A, Bakari M, Mwami J, Sandstrom E (2009). Willingness to volunteer in a Phase I/II HIV vaccine trial: a study among police officers in Dar es Salaam, Tanzania.. Glob Health Action.

[21] Patton MQ (2002). Qualitative Research and Evaluation Methods..

[22] Ruzagira E, Wandiembe S, Bufumbo L, Levin J, Price MA (2009). Willingness to participate in preventive HIV vaccine trials in a community-based cohort in south western Uganda.. Trop Med Int Health.

[23] Airhihenbuwa CO (2007). Healing Our Differences: the Crisis of Global and the Politics of Identity..

[24] UNAIDS (2008). Sub-saharan Africa AIDS epidemic update, regional summary..

[25] Dahlgren L, Emmelin M, Winkvist A (2007). Qualitative Methodology for International Public Health..

